# The Rostral Ventromedial and Lateral Medulla Are the Major Areas Responsive to Lung Cancer Progression among Brainstem Lung-Innervating Nuclei

**DOI:** 10.3390/brainsci12111486

**Published:** 2022-11-02

**Authors:** Mo Chen, Yingfu Jiao, Yumiao Shi, Saihong Xu, Dan Tang, Sihan Chen, Po Gao, Xindi Zhang, Xiaojing Zhao, Mengmeng Cai, Weifeng Yu, Kangjie Xie

**Affiliations:** 1 Graduate School, Wannan Medical College, Wuhu 241000, China; 2 Department of Anesthesiology, Renji Hospital, School of Medicine, Shanghai Jiao Tong University, Shanghai 200127, China; 3 Department of Thoracic Surgery, Renji Hospital, School of Medicine, Shanghai Jiao Tong University, Shanghai 200127, China; 4 Department of Anesthesiology, Nantong First People’s Hospital, Nantong University, Nantong 226001, China; 5 Department of Anesthesiology, The Cancer Hospital of the University of Chinese Academy of Sciences (Zhejiang Cancer Hospital), Research Center for Neuro-Oncology Interaction, Institute of Basic Medicine and Cancer (IBMC), Chinese Academy of Sciences, Hangzhou 310022, China

**Keywords:** lung cancer, brain–lung axis, crosstalk, brainstem nuclei, vagal core, c-Fos, pseudorabies virus, retrograde tracing, neural circuit

## Abstract

In recent years, the information crosstalk between the central nervous system and the periphery has been a hot topic, such as the brain–gut axis, brain–lung axis, etc. Among them, some studies have shown that brainstem nuclei activity can significantly affect the progression of peripheral tumor; however, regarding lung cancer, our understanding of the basic characteristics of the lung-innervating brain nuclei responsive to lung cancer progression remains deficient. Therefore, we used the pseudorabies virus for retrograde labeling of nerves to study the neural circuits between the lung and brain. We then established a mouse orthotopic lung cancer model and used the expression of the c-Fos gene in brain regions to characterize activated brain circuits and compared these results with those of the control group. We focused on c-Fos activity in nuclei associated with retrograde tracing regions of the brainstem. We found over 16 nuclei in the whole brain with direct or indirect lung innervation through neural retrograde labeling with the pseudorabies virus. We further revealed that the neuronal activity of the rostral ventrolateral reticular nucleus (RVL), caudal nucleus of Raphe (raphe obscurus nucleus, ROb), Raphe pallidus nucleus (RPa), and ventral gigantocellular reticular nucleus (GiV) in the rostral ventromedial and lateral medulla were significantly changed in an orthotopic lung cancer mouse model by the immunostaining of c-Fos early responsive protein. Thus, the distinctive rostroventral medulla area, functionally closely related to the vagus nerve, likely plays a role in central neural interaction with peripheral lung tumors and deserves future investigation.

## 1. Introduction

Lung cancer is one of the most common cancers worldwide [1], accounting for approximately 27% of all cancer-related deaths in recent years [2]. Although there are several factors related to the occurrence and progression of lung cancer, including external environmental and internal factors such as smoking, occupational carcinogens, and gene mutations [3], the functional status of the brain, the key modulation system of our body, has rarely been studied in terms of lung cancer regulation [4,5].

The pulmonary innervating neural circuits and neural excitability changes in responsive nuclei are two basic areas that require investigation to clarify the modulation role of the brain in lung cancer. Previously, the distribution of vagal preganglionic neurons of the lung, heart, and stomach have been labeled and compared using non-trans-synaptic tracer horseradish peroxidase [6], and the trans-synaptic tracer pseudorabies virus (PRV) [7] has been used to detect the parasympathetic preganglionic neurons innervating peripheral airways with the elimination of sympathetic related nuclei [8]. In addition, the communication between the central nervous system and the bronchopulmonary system is mainly dominated by the vagus nerve [9,10]. The nucleus of the medulla oblongata can receive information from glutamatergic neurotransmitters in the vagal afferent nerves in the lung, and the central nervous system can participate in the release of acetylcholine through the vagus nerve pathway and binding to the a7 nicotinic acetylcholine receptor (a7nAChR) [11,12] on the surface of lung ILC2 immune cells’ neuroimmune regulation. Sensory neurons in the lung also receive information from neurotransmitters such as calcitonin gene-related peptide, vasoactive intestinal peptide, and neuropeptide Y [4,13]. As further investigations into the pattern of lung-related neural circuits are rare, we aimed to shed light on this issue in our study.

In addition to obtaining neuroanatomical information, it is important to consider the functional interactions between the lungs and the brain. Previous studies have demonstrated extensive communication between the central nervous system and peripheral organs, such as the gut, lung, and others [14,15]. Han et al. found and established that the vagal brain–gut axis is an important component of the neuronal reward pathway by using crosstalk of the neuronal labeling [16]. A paper published in *Nature* in 2022 also revealed the existence of brain–lung axis and proposed that the imbalance of lung microbiota would significantly affect the autoimmune response of the central nervous system [17]. Among them, the brain–gut axis and the brain–lung axis are bidirectional pathways between the central nervous system and the periphery, involving the crosstalk of the neuroanatomical pathway, immune pathway, endocrine pathway, and microbial pathway [18]. The autonomic nervous system is a neuroanatomical pathway for two-way communication between the central and peripheral regions. In the bidirectional network of the brain–lung axis, the neuroanatomical pathways are mainly innervated by the parasympathetic nerves and the sympathetic nerves of the thoracic spinal cord, of which the vagus nerve dominates [19]. When the sensory neurons on the surface of the lung receive external stimulation, the vagal afferent nerves can transmit information to the central nervous system. The information is integrated by the brain and then fed back to the peripheral tissues through the vagal efferent nerves, thereby achieving crosstalk between the brain and the lung. In addition to the neuroanatomical pathway, neuropeptides and other biomarkers are also recognized as important mediators of communication between the central nervous system and peripheral effector cells, and they constitute the neurochemical basis of central and peripheral information crosstalk [18]. In neurodegenerative diseases, we can improve prognosis by monitoring peripheral immune markers to detect related central system diseases early and establish personalized treatment plans [20]. For example, IL-6 can be elevated in the cerebrospinal fluid and peripheral blood of patients with stroke and changes in various chemical substances, oxidase, etc., in the periphery of patients with multiple sclerosis [21]. These demonstrate the importance of central and peripheral neurochemical pathways. However, the roles of these immune cells and biomarkers in the brain–lung axis still need to be further elucidated.

In recent years, there have been related studies on the regulatory role of different brain regions on the periphery. Studies have shown that the ventral tegmental area of the midbrain can regulate tumor growth through the neuroimmunomodulatory pathway [22]. In addition, the right frontal–basal ganglia–thalamic network in the central nervous system is involved in the ability to inhibit hand motor responses, a process that drives the subthalamic nuclei to implement movement inhibition via the hyperdirect pathway [23,24]. The brain, especially the brainstem nuclei, likely affects peripheral tumor incidence and progression [25,26]. As the main center of the vagus nerve, the brainstem is involved in the physiological modulation of different visceral organs and basic vital signs through the sympathetic and parasympathetic nervous systems; for example, the brainstem regulates pulmonary respiratory function [27,28]. Furthermore, the brainstem is an important relay station for ascending and descending transmission when peripheral inflammation or tumors are present [29,30]. Some studies have found that C1 neurons in the rostroventrolateral reticular nucleus (RVL) of colon–adenocarcinoma-bearing mice are activated, and the activity levels of these neurons significantly affect tumor growth in these mice [26], indicating the indispensable role of the brainstem in regulating tumor progression. As the whole brain is complex and functionally, evolutionarily segregated into different sections, we focused on the brainstem to directly investigate the interaction between the autonomic nervous aspect of the CNS and lung tumors. To date, the changes in and functional roles of the brainstem nuclei during lung-cancer-induced stimulation remain under studied, and several questions urgently need to be answered.

Currently, neural-tracing techniques are common research methods used in the field of neuroscience [31]. The pseudorabies virus can readily infect cells of the nervous system and proliferate retrogradely across multiple synapses along the neural circuit [8]; thus, this virus is widely used to retrogradely trace the neural network innervating the peripheral organs. Additionally, c-Fos is an immediate responsive gene in neurons that can be induced by various stimuli and is used to characterize changes in neuronal activation in the brain, acting as a reliable marker of neural activity [32].

This study used the pseudorabies virus for cross-synaptic neural labeling to locate the neurons projecting from the brainstem to the lung. Furthermore, we used c-Fos protein immunofluorescence staining to characterize the activated brainstem regions responsive to lung cancer by comparing lung cancer samples with samples from a non-lung-cancer control group. Finally, the distribution of brainstem nuclei innervating the lung that were responsive to peripheral lung cancer was analyzed synthetically and comprehensively.

## 2. Materials and Methods

### 2.1. Animals

Six to eight week old male, wild-type C57BL/6J mice purchased from Shanghai Jie Sijie Laboratory Animal Co., Ltd. (Shanghai, China), were used in this study. These mice were used to construct orthotopic lung cancer models and for examining viral tracing and c-Fos expression. The animals were housed centrally in standard conditions (temperature, 22 °C; humidity, 40%), with a 12/12 h light/dark cycle. Water and food were provided adequately, and the experimental procedures were conducted in accordance with relevant regulations regarding the ethics of animal experimentation.

### 2.2. Cell Lines

The murine Lewis lung carcinoma (LLC) cell line was purchased from Shanghai FuHeng biological cell bank and was routinely cultured in DMEM high-glucose medium (Gibco) containing 10% fetal calf serum, 100 U/mL penicillin, and 0.01 mg/mL streptomycin at 37 °C in a 5% CO_2_ incubator.

### 2.3. Establishment of the Murine Orthotopic Lung Cancer Model

A total of 10 C57BL/6J mice were randomly divided into the experimental (LLC) and control groups (n = 5 per group). The concentration of tumor cells in suspension was adjusted to 1000 cells/µL. The cells were mixed with Matrigel (Corning) at a 1:1 ratio and refrigerated until injection [33]. The cell mixture was transferred to a 10 µL syringe (Shanghai high pigeon) for injection, and the syringe was loaded onto stereotaxic apparatus.

The mice were anesthetized with pentobarbital (35 mg/kg) solution by intraperitoneal injection. Then, the mice were positioned in the right lateral decubitus position with the left chest facing up, and a small 0.5–1 cm incision was made over the skin just below the scapula. The chest wall muscles were gently spread until the intercostal space and pleura were clearly visible, and then the ribs were counted from the lower border of the thorax upward. The tumor cells were injected between the fourth and fifth ribs at the posterior axillary line. At the time of inoculation, the syringe was slowly advanced 3.5 mm into the lung parenchyma, and 2 µL of the tumor cell mixture was injected vertically at an optimal angle. After injection, the needle was left in place for 5–10 s, and the incision was sutured with 1–2 stitches. The mice were subsequently administered analgesic medication and allowed to recover in a prewarmed incubator for 20 to 30 min. For the control group, the same method described above was performed with an equivalent volume of 0.9% saline injected into the left lung of each mouse. The mice were then housed for three weeks; subsequently, perfusion fixation was performed under deep anesthesia, and brain tissue samples were taken.

### 2.4. Pulmonary Orthotopic Injection of Pseudorabies Virus

The (P03001 PRV-CAG-EGFP, Wuhan axial) injection method for PRV was generally consistent with the tumor inoculation method. One microliter of PRV was injected into each of the upper, middle, and lower three sites in the left lobe of the mouse, and the syringe was left in place for approximately 1 min after injection to prevent viral extravasation. The incision was sutured; the mice were housed in an SPF-grade environment after resuscitation and observed for survival status. When mice were in poor condition, they were anesthetized and perfusion-fixed, and brain tissues were taken.

### 2.5. Perfusion

Mice were deeply anesthetized with pentobarbital (35 mg/kg) and transcardially perfused with 20 mL of 0.9% saline followed by 20 mL of 4% paraformaldehyde (PFA). Brains were extracted and fixed in 4% paraformaldehyde overnight, and then dehydrated by gradient sedimentation in 20% and 30% sucrose; samples were stored at 4 °C until sectioning.

### 2.6. Immunofluorescence Staining

Brains were sectioned coronally at 30 µm thickness with a freezing microtome. One out of every four brain sections was collected in a 0.1 M phosphate-buffered saline (PBS) [34]. Standard immunofluorescence staining for c-Fos was performed on brain sections from selected brainstem sites. The sections were rinsed three times in PBS on a shaker for 8 min per rinse. The sections were incubated in PBS containing 1% Triton-X 100 for 30 min and rinsed again in PBS containing 0.3% Triton-X 100 for 5 min. After rinsing, the sections were blocked with an immunostaining blocking solution (Beyotime) for 1 h. Sections were then incubated in a rabbit anti-c-Fos primary antibody solution (Abcam, dilution 1:800) for 48 h at 4 °C. Then, the sections were rinsed in PBS on a shaker for 10 min per rinse and incubated with an Alexa Fluor 488-conjugated donkey-anti-rabbit secondary antibody solution (Abcam, dilution 1:800) for 1.5 h at room temperature. Finally, the sections were rinsed three times in PBS on a shaker for 10 min per rinse and counter stained with DAPI (SouthernBiotech). If a represented section of an area was lost during sectioning, the sample was excluded from the study. Therefore, our sample numbers varied slightly between different brain areas, and the sample number per brain region was less than 5 (n ≤ 5).

### 2.7. Data and Statistical Analysis

Immunolabeled tissues were analyzed and imaged using the OLYMPUS digital slide scanning system and OLYVIA software and Adobe Photoshop software was used for analyses. The sliced images were superimposed with the corresponding Atlas maps to determine the different brain regions. Once a brain region was determined, Fos-immunopositive neurons in that region were counted manually using the Image J counting tool. Finally, the mean value was calculated as the final number of Fos-immunopositive neurons in that brain region (total number of Fos-immunopositive neurons in that brain region/number of slices in that brain region). Statistical analyses (Table 1) were performed using Prism 8 software and a two-tailed independent samples *t*-test was used to assess between-group differences in c-Fos-immunopositive neuron counts, with *p* values < 0.05 considered to indicate significance [35].

## 3. Results

### 3.1. Retrograde Labeling of Lung-Associated Nuclei by Pseudorabies Virus

**Figure 1:**
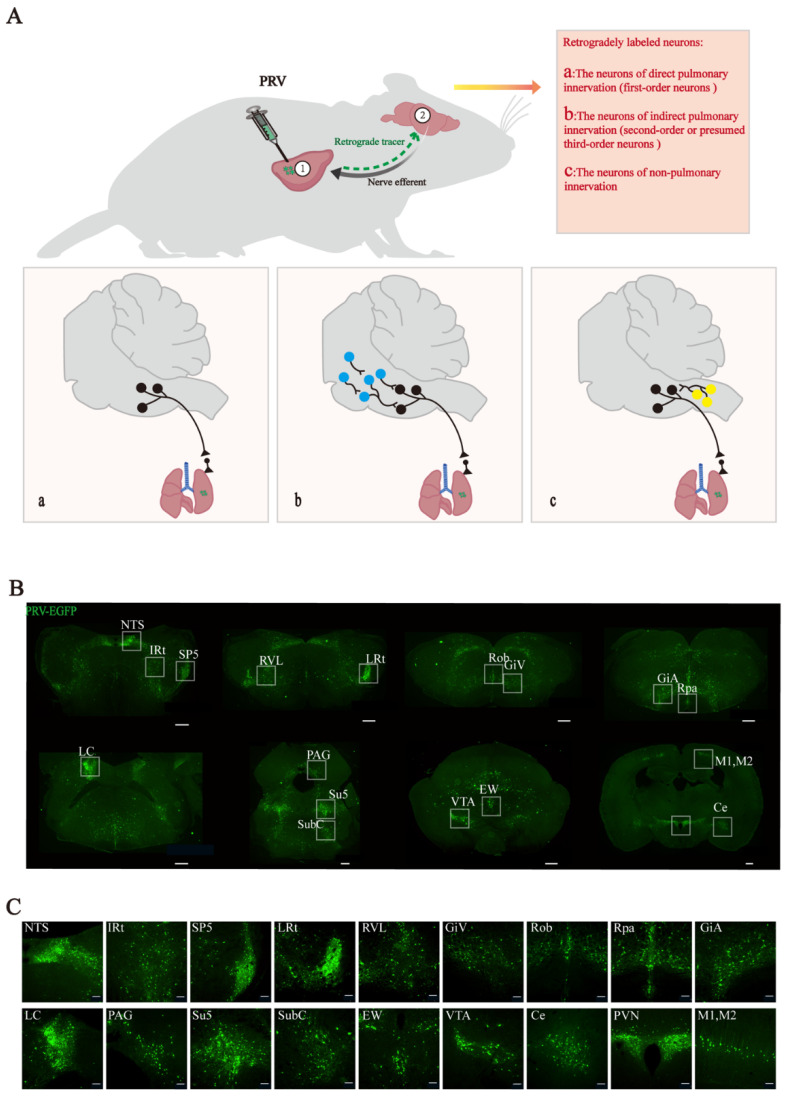
Schematic diagram of retrograde labeling of pseudorabies virus lungs and virus tracer-positive brain regions. (**A**) In situ injection of pseudorabies virus in the left lung of mice. The neuronal projections between retrogradely labeled neurons and lung tissue can be divided into direct pulmonary innervating neurons (**a**), indirect pulmonary innervating neurons (**b**), and nonpulmonary innervating neurons (**c**). (**B**) Overview of coronal sections of pseudorabies virus retrogradely labeled brain regions (scale bar = 400 μm). (**C**) Enlarged brain sections retrogradely labeled by pseudorabies virus (scale bar = 100 μm). NTS = nucleus tractus solitarius, IRt = intermediate reticular nucleus, SP5 = spinal trigeminal nucleus, LRt = lateral reticular nucleus, RVL = rostroventrolateral reticular nucleus, GiV = gigantocellular reticular nucleus, ventral part, ROb = Raphe obscurus nucleus, RPa = Raphe pallidus nucleus, GiA = gigantocellular reticular nucleus, alpha part, LC = locus coeruleus, PAG = periaqueductal grey, Su5 = supratrigeminal nucleus, SubC = subcoeruleus nucleus, EW = Edinger–Westphal nucleus, VTA = ventral tegmental area, M1,M2 = primary, secondary motor cortex, PVN = paraventricular hypothalamic nucleus, and Ce = central amygdaloid nucleus.

The brain-to-lung axis has long been our research focus. To further explore the neural connection between the lung and the central nervous system, we injected PRV into the left lung parenchyma of mice and found retrograde labeled neurons in the corresponding brain regions. To study the relationship between labeled neurons and lung tissue, we roughly classified these neurons and generated a schematic diagram (Figure 1A). According to the projection relationship between nuclei and lung tissue, PRV-labeled neurons were divided into three categories. Primary pulmonary innervation neurons, which directly retrogradely project from the lung tissue and downwardly regulate lung tissue (Figure 1(Aa)); secondary or tertiary pulmonary innervation neurons, which indirectly retrogradely project from the lung tissue and downwardly regulate lung tissue (Figure 1(Ab)); and nonpulmonary innervation neurons, which project retrogradely but are not involved in the downward regulation of lung tissue (Figure 1(Ac)).

After PRV lung injection, more nerve nuclei were retrogradely labeled by PRV in the medulla oblongata. These include NTS, IRt, LRt, SP5, RVL, ROb, RPa, and GiV. A few nuclei labeled in the pons, such as LC.PAG, Subc, Su5, and EW, were also retrogradely labeled by PRV. There were also a few retrogradely labeled neurons in the VTA of the hypothalamus and the PVN. Finally, PRV retrogradely labeled other regions, including M1, M2, and Ce (Figure 1B).

### 3.2. Activation of c-Fos in Lung-Associated Nuclei in the Brainstem Region

**Figure 2:**
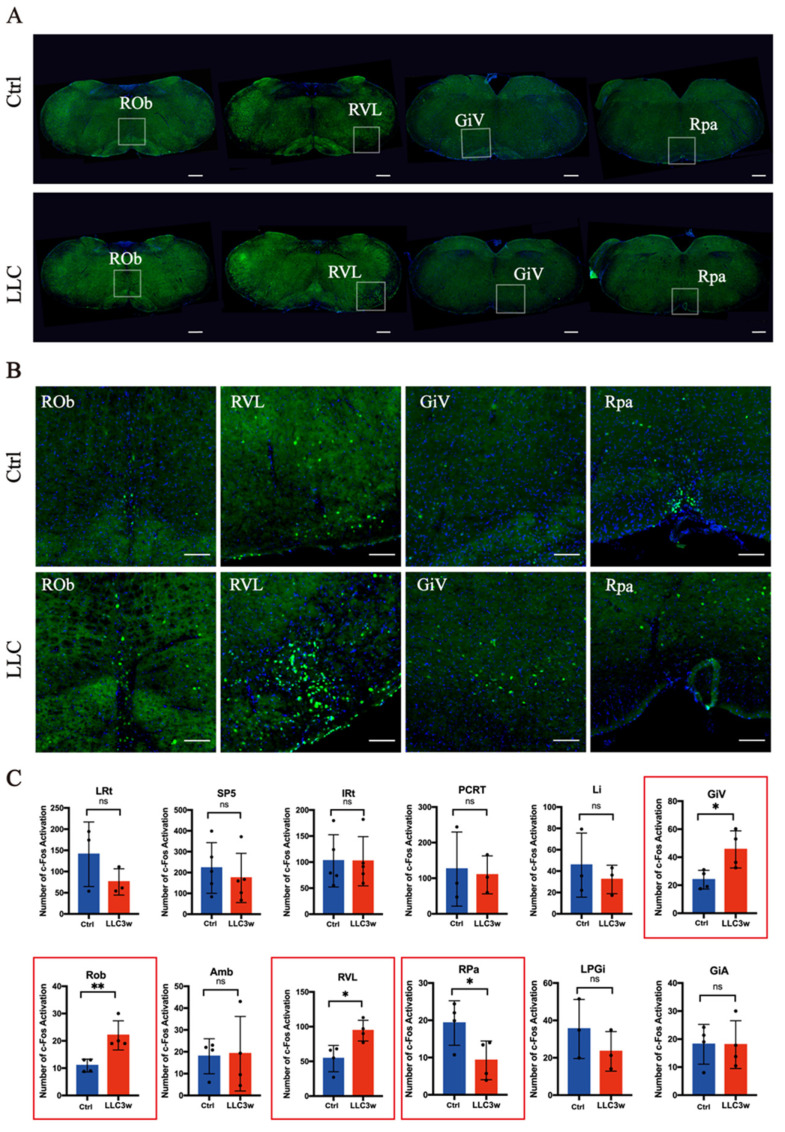
Overall c-Fos activation patterns in the medulla oblongata. (**A**) Overview of coronal sections in the medulla oblongata of LLC and control mice (scale bar = 400 μm). (**B**) Enlarged photomicrographs of the medulla oblongata regions illustrate c-Fos activation in various brain structures of LLC and control mice (scale bar = 100 μm). (**C**) Levels of c-Fos-activated neurons in LLC and control groups. There are significant differences in GiV (*p* < 0.05), ROb (*p* < 0.01), RVL (*p* < 0.05), and RPa (*p* < 0.05) between the LLC group and the control group (*p* < 0.05). PCRt= parvicellular reticular nucleus, Li = linear nucleus of the medulla, Amb = ambiguus nucleus, and LPGi = lateral paragigantocellular nucleus. “*” represents *p* < 0.05, “**” represents *p* < 0.01, “ns” represents no significant differences, red bars represent the experimental group (LLC3w) and blue bars represent the control group (Ctrl). Statistically different nuclei are represented in the red box.

**Figure 3:**
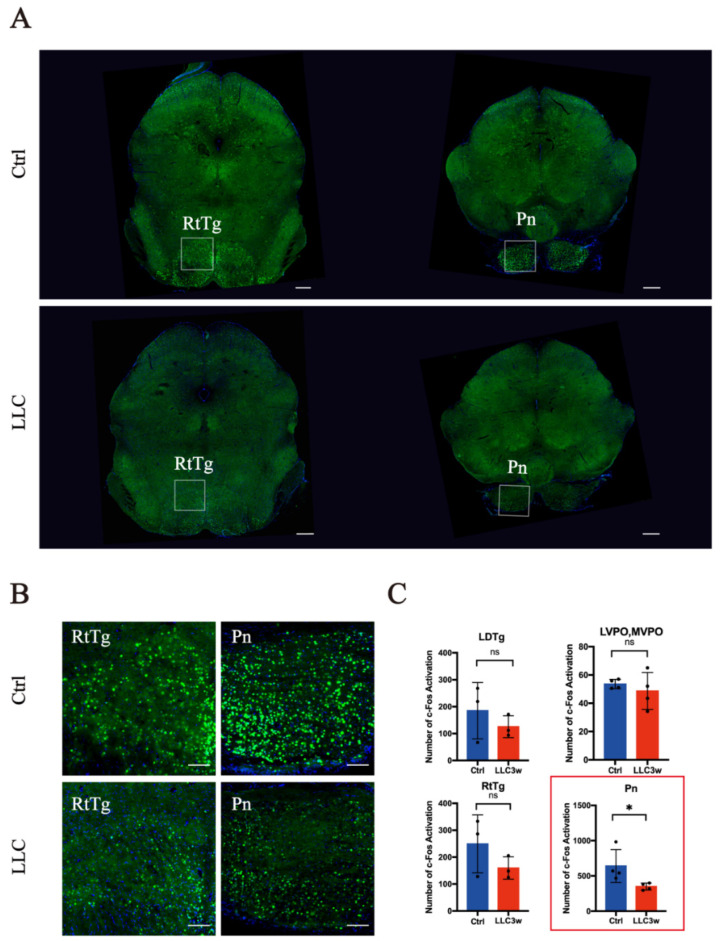
Overall c-Fos activation patterns in the pons. (**A**) Overview of coronal sections in the pons of LLC and control mice (scale bar = 400 μm). (**B**) Enlarged photomicrographs of the pontine regions illustrate c-Fos activation in various brain structures of LLC and control mice (scale bar = 100 μm). (**C**) Levels of c-Fos-activated neurons in LLC and control groups. There is a statically significant difference in Pn (*p* < 0.05) between the LLC group and the control group (*p* < 0.05), but there is no statically significant difference in LDTg, LVPO, MVPO, and RtTg between the LLC group and the control group (*p* > 0.05). LDTg = laterodorsal tegmental nucleus, LVPO, MVPO = lateroventral periolivary nucleus, medioventral periolivary nucleus, RtTg = reticulotegmental nucleus of the pons, and Pn = pontine nuclei. “*” represents *p* < 0.05, “ns” represents no significant differences, red bars represent the experimental group (LLC3w) and blue bars represent the control group (Ctrl). Statistically different nuclei are represented in the red box.

**Figure 4:**
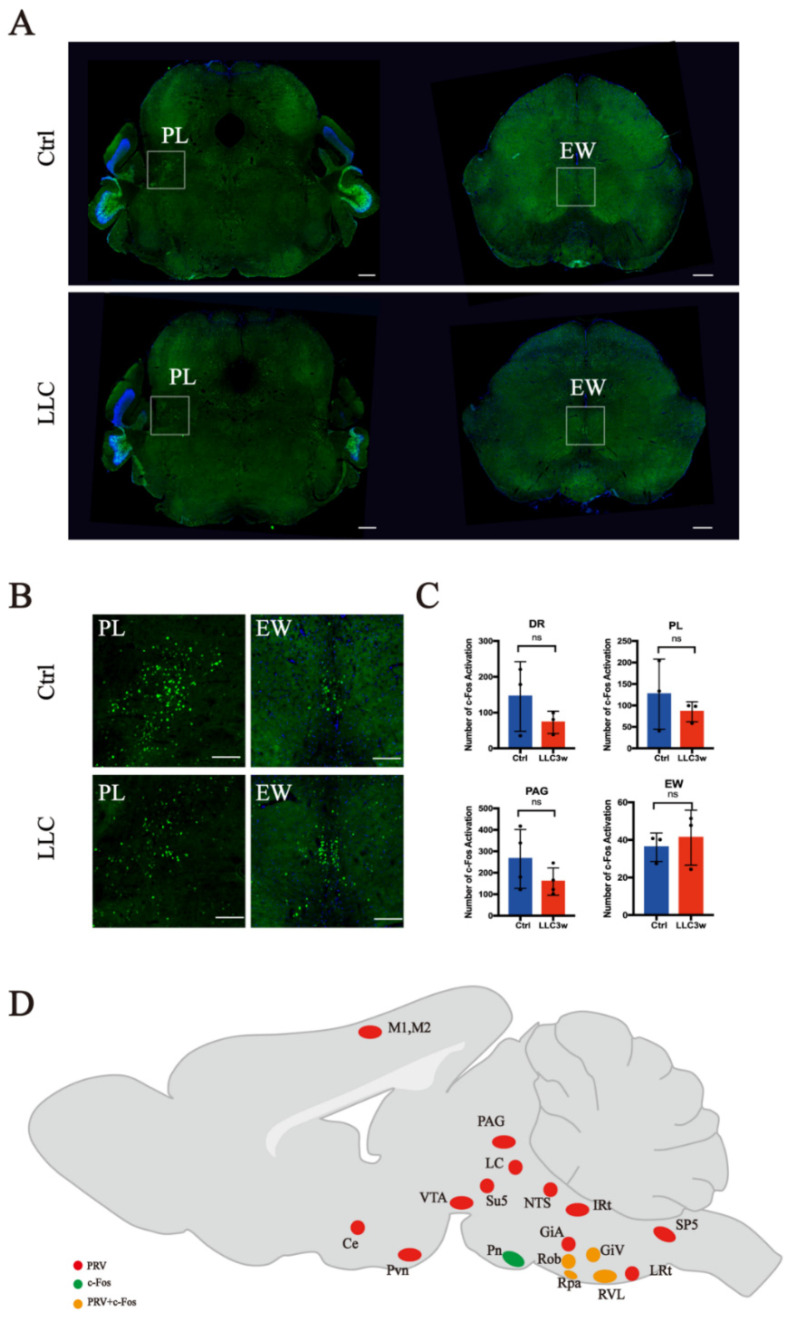
Overall c-Fos activation patterns in the midbrain. Schematic diagram of the overlap of PRV-positive and c-Fos-positive brain regions. (**A**) Overview of coronal sections in the midbrain of LLC and control mice (scale bar = 400 μm). (**B**) Enlarged photomicrographs of the midbrain regions illustrate c-Fos activation in various brain structures of LLC and control mice (scale bar = 100 μm). (**C**) Levels of c-Fos-activated neurons in LLC and control groups. There is no statically significant difference in DR, PL, PAG, and EW between the LLC group and the control group (*p* > 0.05). DR = dorsal Raphe nucleus, PL = paralemniscal nucleus. (**D**) Schematic diagram PRV and c-Fos co-labeled brain regions. Red represents neurons retrogradely labeled with PRV, green represents neurons with changes in c-Fos activation in orthotopic lung cancer, and orange represents neurons co-labeled with c-Fos and PRV. “ns” represents no significant differences, red bars represent the experimental group (LLC3w) and blue bars represent the control group (Ctrl).

To further study the brain circuits in the lung cancer model, combined with the results of retrograde lung tracing, we used c-Fos to characterize the brainstem regions activated in lung cancer. C-Fos is an inducible gene activated by various stimuli and is a reliable marker of neural activity. The number of c-Fos-positive neurons in the brainstem of lung cancer mice decreased overall (Figure 2, Figure 3 and Figure 4 show the expression of c-Fos in the experimental and control groups, respectively). We focused on the anatomical structure of the mouse brain and counted the number of c-Fos positive neurons in the medulla oblongata (Figure 2), pons (Figure 3), and midbrain (Figure 4). The neurons with significant changes in c-Fos expression in the nuclei retrogradely labeled by PRV were mainly distributed in the RVL, ROb, RPa, and GiV. In addition, the expression of c-Fos in the Pn, which PRV did not retrogradely label, also changed significantly. In the nuclei with c-Fos changes, RVL mainly participates in the transmission of information required for the peripheral stimulus response. Pn is an important relay station for receiving information from the brain, while GiV, ROb, and RPa are mainly involved in the output of downward regulatory information.

In the medulla oblongata of the brain stem, c-Fos was highly expressed in the RVL, ROb, and GiV of the experimental group (Figure 2A,B). In the control group, the expression of c-Fos in the RPa was higher (Figure 2A,B). In the medulla oblongata, there was a significant difference in the expression of c-Fos between the experimental and control groups (RVL *p =* 0.0156, ROb *p =* 0.0092, GiV *p =* 0.0271, and RPa *p =* 0.0450) (Figure 2C). In the pons, the expression of c-Fos in the Pn was higher in the control group (Figure 3A,B) than in the experimental group (Figure 3A,B), and there was a statistical difference between the two groups (Pn *p =* 0.0492, Figure 3C). However, there was no significant difference in the expression of c-Fos in the related nuclei in the midbrain (Figure 4A–C).

To explore the activation of lung-associated nuclei in the lung cancer model, we overlapped the results of PRV retrograde tracing with the nuclei with statistically different c-Fos-positive expression levels and constructed a diagram of the co-labeled areas. We found that the GiV, ROb, RPa, and RVL of the medulla oblongata exhibited PRV retrograde labeling and c-Fos-positive expression (Figure 4D). Our results suggest that these nuclei may be involved in the neural regulation of lung tissue protection under tumor conditions.

## 4. Discussion

In the present study, we systematically described the distribution of all nuclei in the brain with a neural projection relationship with the lung and found that the RVL, RPa, ROb, and GiV in the rostroventral medulla region were distinctively responsive to lung tumor progression. The RVL, GiV, and ROb were activated and the RPa was relatively suppressed; thus, these nuclei are likely involved in the neural feedback regulation of lung tumors. Our results briefly characterize the possible interaction between the brainstem nuclei and peripheral lung tumors.

Traditionally, the RPa, ROb, GiV, and RVL are not considered the main functional regulation regions for the lung. The RPa, ROb, and GiV are the major components of the RVM, which is recognized as the area involved in the downstream regulation of pain, while RVL is an important region involved in regulating cardiovascular activities [36,37]. However, there is some evidence to suggest that these nuclei are also involved in respirational regulation; for example, as one of the two serotoninergic nuclei in the whole brain, the serotonergic neurons in the Raphe nuclei of RVM have been previously implicated in respiratory networks [38,39,40,41], while stimulation of the catecholaminergic neurons (C1 neurons) in the RVL has been shown to increase the respiratory rate [42,43,44].

Moreover, other visceral regulation functions for these nuclei have been found. GiV is known to be a cardiac inhibitory brain region independent of the higher center, and stimulation of this region can lead to hypotension and bradycardia [45,46]. The catecholaminergic neurons and glutamatergic neurons in the RVL participate in the central regulation of infection, inflammation, and hypoxia by innervating sympathetic and parasympathetic preganglionic neurons [26,36,47,48,49]. The specific activation of glutamatergic neurons in the RVL can drive intestinal sympathetic nerve activity and regulate gastrointestinal peristalsis [50,51]. These findings represent the continuously improved knowledge of the functions of these nuclei; thus, it is worth investigating the RVM and RVL further to determine their role in lung tumor progression.

Although Pn is not lung-innervating, the neuronal activity of Pn was suppressed in the lung cancer model. As an important motor information transmission station between the cerebral cortex and the cerebellum [52,53], Pn receives extensive projections from the reticular formation of the brainstem, LC, PAG, and the Raphe nucleus [54]. Therefore, we speculate that during lung cancer progression, the neural activity changes in the Raphe nucleus may indirectly affect the pontine nucleus to initiate functional coordination.

To date, it remains unclear how lung tumors communicate with the corresponding neural nuclei in the brainstem and how the activities of related neurons affect lung tumor progression. Previous studies have shown that the brain can innervate tumors or peripheral immune organs through sympathetic or parasympathetic nerve fibers [55,56,57]. Specific neuron types, such as the mouse vagal afferent p2ry1 neurons, have recently been identified. These neurons are fast-conducting A fibers that interact with pulmonary endocrine cells and activate p2ry1 neurons, significantly inhibiting respiration without affecting other visceral autonomic nerve functions [27]. Regarding the possible interaction mode, a paper published in *Nature* in 2022 revealed the existence of the lung-to-brain axis and proposed that an imbalance in the lung microbiota could significantly affect the autoimmune response in the CNS [17]. The “inflammatory reflex” [58] describes another possible interaction mode between the lung and brain in which the CNS responds to peripheral inflammatory stimuli and modulates the inflammatory response reflexively [59,60]. At every stage of tumor progression, changes in the tumor microenvironment, such as release of cytokines and other inflammatory substances around the tumor, stimulate the corresponding afferent nerves and send information to the brain. The responsive nuclei will integrate this information and mobilize multiple systems of the body to respond to the peripheral changes [61,62,63]. Detailed and specific information regarding lung tumors remains lacking.

This study has some limitations. Although it has been reported that the expression level of c-Fos protein increases throughout chronic stimulation, such as in chronic social stress situations, lung tumor progression may not cause long-lasting changes in neurons, and c-Fos protein expression alone is insufficient to describe neuronal activity changes in the lung cancer model. The neuro–tumor interaction mechanism between neuronuclei and lung tumors is complex, and this study reveals the activation of nuclei during tumor burden in lung cancer, and further exploration of its mechanism is needed. In addition, we did not specifically identify which nuclei in the ventral medulla are more important for lung tumor progression, and the regulatory mechanisms of these nuclei on lung function have not been investigated. Previous studies have shown that the medullary vagal efferent nerves can participate in neuroimmunoregulation by releasing acetylcholine to peripheral lung tissue [18]; however, whether these neurotransmitters affect lung tumors remains to be verified.

Once we discover the types of neurons in the ventral medulla nuclei that communicate with peripheral lung tumors, and how these neurons interact with the immune system, they will be prospects for regulating tumor growth by regulating neurotransmitters or neurohormones or the activity of neuronal nuclei.

## 5. Conclusions

Our study provided a whole picture of the central neural network innervating the lung directly or indirectly, and by further focusing on the neuron activity changes in the brainstem, we revealed that the medulla oblongata (NTS, IRt, LRt, SP5, RVL, ROb, RPa, and GiV), pons (LC.PAG, Subc, Su5, and EW), midbrain, thalamus, and cerebral cortex (M1, M2, and Ce) all contain nerve nuclei that have a direct neural connection to the lungs. The expression of c-Fos in GiV, ROb, and RVL brain regions of mice with lung cancer was upregulated, while the expression of c-Fos in RPa and Pn brain regions was decreased. RVL, ROb, RPa, and GiV in the ventral medulla oblongata are apparently important for CNS-associated lung tumor regulation.

These results provide basic information regarding brain and lung tumor interaction, and the rostroventral medulla is likely an important region for CNS-associated lung tumor regulation. In the future, we need to further clarify the types of rostroventral medulla neurons, and use techniques such as intracerebral stereotaxic injection, immunofluorescence, and electrophysiology to regulate the neurons in these nuclei to observe the effect of these neurons on lung tumor progression.

## Figures and Tables

**Table 1:** c-Fos statistics of brainstem nuclei.

	**LRt**	**SP5**	**IRt**	**PCRT**	**Li**	**GiV**	**ROb**	**Amb**	**RVL**	**RPa**
Number of mice	3	5	5	3	3	4	4	4	4	4
*p* value	0.2414	0.5443	0.9815	0.8220	0.5186	0.0271	0.0092	0.9060	0.0156	0.0450
	**LPGi**	**GiA**	**LDTg**	**LVPO, MVPO**	**RtTg**	**Pn**	**DR**	**PL**	**PAG**	**EW**
Number of mice	3	4	3	4	3	4	3	3	4	3
*p* value	0.3363	0.9806	0.4133	0.4963	0.2493	0.0492	0.2872	0.4515	0.2123	0.6226

A two-tailed independent samples *t*-test was used to assess between-group differences in c-Fos-immunopositive neuron counts, with *p* values < 0.05 considered to indicate significance.

## Data Availability

Not applicable.
